# Sequential reaching in older adults: vibrotactile feedback improves preparation and movement control

**DOI:** 10.3389/fnagi.2026.1840948

**Published:** 2026-07-17

**Authors:** Saba Mohammadalinezhad Kolahdouz, Quinn Malone, Steven R. Passmore, Jonathan J. Marotta, Cheryl M. Glazebrook

**Affiliations:** 1 Perceptual Motor Integration Lab, Applied Health Sciences, Faculty of Kinesiology and Recreation Management, University of Manitoba, Winnipeg, MB, Canada; 2 Sensorimotor Physiology and Integrative Neuromechanics, School of Health and Exercise Sciences, Faculty of Health and Social Development, University of British Columbia, Okanagan, Kelowna, BC, Canada; 3 Perceptual Motor Behavior Lab, Faculty of Kinesiology and Recreation Management, University of Manitoba, Winnipeg, MB, Canada; 4 Perception & Action Lab, Psychology, Faculty of Arts, University of Manitoba, Winnipeg, MB, Canada

**Keywords:** aging, audio feedback, augmented feedback, motor activity, vibration

## Abstract

**Introduction:**

Age-related motor decline is particularly evident during complex movements that require sequencing and bimanual coordination. The one-target advantage (OTA), in which single-target movements are typically initiated faster than the first segment of a two-target sequence, provides a useful framework for assessing planning demands in older adults.

**Methods:**

We examined whether task complexity and brief auditory or vibrotactile feedback delivered at the first target contact influence sequential reaching performance in 24 older adults (67.46 ± 5.41 years). Participants completed one- and two-target reaching tasks under three feedback conditions (no feedback, auditory feedback, or vibrotactile feedback) across movement types varying in complexity, including extension, reversal, unimanual, and bimanual movement sequences. Outcome measures included movement preparation, movement execution, movement transitions, kinematic measures, and endpoint accuracy.

**Results:**

Vibrotactile feedback selectively improved performance by shortening reaction time in the unimanual extension task relative to one-target movements, effectively reversing the typical OTA pattern. Vibrotactile feedback also increased peak velocity and altered kinematic control during the second movement segment. In contrast, movement time to the first target remained unchanged, whereas movement time to the second target increased with task complexity and was longest during bimanual conditions. Endpoint accuracy and consistency remained stable across feedback and task conditions, indicating that the kinematic benefits of vibrotactile feedback did not occur at the expense of either endpoint accuracy or endpoint consistency.

**Discussion:**

Overall, brief vibrotactile cues enhanced movement preparation and kinematic control in older adults during sequential reaching, particularly under higher coordination demands.

## 1 Introduction

Aging is associated with systematic changes in motor control that influence how movements are planned, executed, and adjusted through sensory feedback (Seidler et al., 2010; Goodman and Tremblay, 2021; Mohammadalinezhad Kolahdouz, et al., 2026 a; Yazdani et al., 2020a). These changes arise from reduced efficiency across sensorimotor systems, including proprioceptive, visual, and vestibular processes, which contribute to slower and more variable goal-directed actions (Seidler et al., 2010; Poirier et al., 2020). Age-related motor deficits become particularly evident in tasks requiring rapid online corrections or coordination across multiple effectors (Gómez-Granados et al., 2021; Li et al., 2023). Within this context, motor performance in older adults has been linked to differences in processing speed, proprioceptive processing, and movement coordination, which may limit the efficiency of movement planning and execution, particularly during tasks requiring multiple movement segments, directional reversals, or coordination between limbs (Wang et al., 2020).

Sequential target aiming provides a well-established framework for examining how planning and execution interact during goal-directed movement. A central finding within this paradigm is the one-target advantage (OTA), which refers to faster performance when reaching to a single target compared to the first segment of a two-target sequence (Adam et al., 2000). The OTA has been consistently observed across variations in limb use, practice, and feedback conditions, suggesting that it reflects central planning processes rather than peripheral constraints (Khan et al., 2010; Lawrence et al., 2013, 2016). These findings support the view that sequential movements are organized through integrated planning mechanisms that span multiple movement segments. The Multiple Process Model of goal-directed aiming provides a useful framework for interpreting these effects because it distinguishes between processes related to movement preparation, initial impulse regulation, and late limb-target control (Elliott et al., 2010, 2017).

However, the expression of sequential movement performance is influenced by task complexity, and aging may alter how these demands are managed. Movements involving directional reversals or switching limbs impose additional demands on inhibitory control, interhemispheric coordination, and sensorimotor planning. Under such conditions, older adults exhibit greater movement variability and longer execution times compared to younger adults (Zvornik et al., 2024). In addition, older adults tend to decompose continuous movements into more segmented components when task demands increase, reflecting a shift toward more cautious, stepwise control strategies (Sarlegna, 2006). Based on these findings, in addition to the expected changes in overall performance, older adults may experience changes in the organization of sequential movement control under increased task demands. One important source of increased task complexity involves movements requiring coordination between limbs. Previous OTA research has demonstrated that switching hands during sequential aiming alters the integration between movement segments and may engage distinct central and peripheral control processes (Khan et al., 2010; Lawrence et al., 2016). In particular, bimanual sequential movements require transitions between effectors and increased interlimb coordination, which may place greater demands on movement preparation and online control, especially in older adults (Zvornik et al., 2024). Because augmented sensory feedback may support movement regulation differently depending on coordination demands, comparing unimanual and bimanual task configurations may provide insight into how sensory augmentation influences sequential motor control under varying levels of interlimb complexity.

Augmented sensory feedback has been proposed as a potential method for mitigating age-related motor deficits (Sigrist et al., 2013; Pomplun et al., 2022). Supplementary sensory information, such as vibrotactile or auditory cues, can enhance movement performance by supporting online movement corrections and reducing reliance on degraded intrinsic feedback. Vibrotactile feedback has been shown to reduce trajectory error and improve motor learning during upper-limb movements (Bark et al., 2014), as well as enhance fine motor control in older adults (Hsu et al., 2021). Similarly, auditory feedback has been associated with improvements in movement timing coordination, and movement efficiency across a range of motor tasks (Ghai et al., 2018; Guo et al., 2022; Yazdani et al., 2020a; Yazdani et al., 2020b). In these contexts, auditory cues typically act as external temporal signals that facilitate movement synchronization and timing. However, unlike vibrotactile feedback, which provides limb-specific somatosensory information directly associated with the moving effector, auditory feedback is less spatially specific and may primarily influence temporal and rhythmic aspects of movement rather than limb-target regulation during discrete reaching tasks. This distinction is also consistent with the Multiple Process Model of goal-directed aiming, which proposes separable contributions of movement preparation, impulse control, and late limb-target regulation during sequential actions (Elliott et al., 2010, 2017).

Auditory feedback was therefore included as a comparison condition to determine whether any improvements in sequential reaching performance were specific to somatosensory augmentation or could also emerge from a more general external sensory cue. Previous work comparing sensory modalities suggests that auditory feedback primarily influences movement timing and coordination (Liang et al., 2024), whereas vibrotactile feedback may provide more movement-specific sensorimotor information linked to body position and online movement regulation. In addition, recent vibrotactile rehabilitation studies have emphasized that tactile feedback may directly supplement proprioceptive and somatosensory processing during motor tasks, particularly in older adults and rehabilitation settings (Guo et al., 2022). Therefore, we predicted that auditory feedback would primarily influence temporal aspects of movement preparation and execution relative to the no-feedback condition, such as reaction time and movement timing, whereas vibrotactile feedback would produce stronger effects on movement-specific kinematic regulation and terminal control. Together, these findings highlight the potential of augmented sensory feedback to support motor control, particularly in aging populations. Recent work suggests that augmented sensory feedback may also influence the organization of sequential movements. In our previous study with younger adults, vibrotactile feedback selectively enhanced late-phase control during sequential reaching without compromising endpoint accuracy, indicating a targeted effect on kinematic regulation rather than a uniform change in movement timing (Mohammadalinezhad Kolahdouz et al., 2026b). In a related study including both younger and older adults, vibrotactile feedback reduced reaction time and, in some cases, altered the typical OTA pattern, while also improving movement time, endpoint accuracy, and deceleration-phase control, particularly in older adults (Mohammadalinezhad Kolahdouz et al., 2026a). These findings suggest that the expression of sequential movement costs, including the OTA, is flexible and can be modulated by sensory context, with older adults showing greater sensitivity to augmented feedback.

Despite this growing body of work, less is known about how sequential movement organization in older adults is influenced by increasing task complexity and augmented feedback. Previous studies have primarily examined OTA and sensory feedback effects in relatively simple, unimanual task configurations. It remains unclear whether these effects generalize to more complex sequential actions involving directional reversals and bimanual coordination, which impose greater demands on planning, coordination, and online control processes. In the present study, movement complexity was operationally defined by the coordination and planning demands associated with the movement sequence. Complexity increased when tasks required multiple movement segments, directional reversals, or coordination between limbs, with bimanual and reversal conditions representing greater sequential control demands than simple unimanual extension movements. Therefore, the present study examined how auditory and vibrotactile feedback influence sequential reaching performance in older adults under varying levels of task complexity. By extending previous work to include more complex task configurations involving extension, reversal, unimanual, and bimanual movement sequences, we aimed to determine whether sensory feedback effects observed in simpler tasks generalize to conditions that place greater demands on motor planning and coordination. Based on prior findings, we hypothesized that task complexity would increase preparation and execution demands, reflected by longer reaction time, pause time, movement time, and altered second-segment kinematics in reversal and bimanual tasks. We further hypothesized that auditory feedback would primarily influence temporal aspects of performance, whereas vibrotactile feedback would produce stronger effects on movement execution and terminal control, particularly peak velocity and time after peak velocity. Finally, we expected that any feedback-related improvements in timing or kinematics would occur without major changes in endpoint accuracy or endpoint variability.

## 2 Method

### 2.1 Participants

An *a priori* power analysis was conducted using G*Power 3.1.9.7 (Faul et al., 2007) to determine the required sample size for the present repeated-measures design. The analysis was informed by effect sizes reported in our prior sequential aiming study using the One-Target Advantage (OTA) paradigm, which demonstrated medium-to-large within-subject effects across temporal and kinematic variables (partial η^2^ ranging from approximately 0.13 to 0.54 across key outcomes, with larger effects observed for movement sequencing measures; Mohammadalinezhad Kolahdouz et al., 2026b). These estimates were also broadly consistent with previous OTA studies using similar sequential aiming paradigms and comparable sample sizes, which reported medium-to-large effects across reaction time and movement timing measures (Khan et al., 2010; Lawrence et al., 2016). Using a repeated-measures ANOVA (within factors) framework with *α* = 0.05, desired power (1 − *β*) = 0.80, and a conservative medium effect size estimate (Cohen’s *f* = 0.25), the required sample size was estimated to range between 18 and 22 participants, depending on the assumed correlation among repeated measures. To ensure adequate power across multiple dependent variables and to account for potential data exclusions, twenty-four right-handed neurotypical older adults (15 females; 9 males; 67.46 ± 5.41 years old) were recruited for the experiment. All participants reported normal or corrected-to-normal vision and hearing had no recent upper limb injuries and were free from neurological or musculoskeletal conditions that could interfere with reaching performance. Written informed consent was obtained from all participants prior to enrollment. The study was approved by the University of Manitoba Research Ethics Board (protocol #HE2024-0286) and conducted in accordance with the Declaration of Helsinki (2013 revision) World Medical Association (2013).

### 2.2 Apparatus

Participants used two styli instrumented for motion tracking. The device was based on a commercially available capacitive stylus (Penyeah 4-in-1 Stylus Pen; 14.2 cm in length, 0.9 cm in diameter, base mass = 21 g). For kinematic recording, a single infrared-emitting diode (IRED) marker was attached approximately 1.2 cm from the distal end of the stylus. The marker was placed along the right lateral surface of the stylus shaft to maintain clear optical visibility during grasp and movement. For trials involving vibrotactile feedback, a miniature brushless DC vibration motor was integrated into the stylus body. The actuator was positioned approximately 2.9 cm proximal to the stylus tip (measured from the contact surface of the disc tip) and mounted on the lateral surface opposite the IRED. Locating the actuator and optical marker on opposing sides of the stylus minimized mechanical interference between vibration and optical tracking while maintaining comfortable hand positioning. The total mass of the stylus assembly, including the IRED and vibration actuator, remained within the typical range of common writing instruments (approximately 23–25 g), minimizing potential effects of device weight on natural reaching movements. All participants performed the task using the same instrumented stylus configuration to maintain consistency across experimental conditions. Three-dimensional movement data were recorded at 300 Hz using an Optotrak 3D Investigator motion capture system (Northern Digital Inc., Waterloo, Canada). Participants performed the task on a 22-inch Dell touchscreen monitor (model E2222H), positioned horizontally on a table at approximately 76 cm height. Experimental control, including stimulus presentation, trial timing, and feedback delivery, was implemented using custom scripts in E-Prime 3.0 (Psychology Software Tools, Sharpsburg, PA, USA), with synchronization achieved via a Chronos response box to ensure precise temporal alignment with movement events.

Visual targets consisted of yellow squares (2.5 × 2.5 cm) displayed on the touchscreen. Although the targets were large enough to permit comfortable contact with the stylus, endpoint coordinates were recorded continuously, allowing endpoint error to be quantified within the target area. Thus, constant and variable error reflected the accuracy and consistency of endpoint location rather than simply whether the participant contacted the target. These were arranged in three vertically aligned pairs along the participant’s midline. Within each pair, targets were separated horizontally by 35 mm (center-to-center), and adjacent pairs were spaced 150 mm apart vertically. This layout was based on established one-target advantage paradigms (e.g., Lawrence et al., 2016; Khan et al., 2010), which reliably induce sequential planning demands while maintaining high accuracy. Two home positions (one for each hand) were presented at the start of each trial.

### 2.3 Sensory feedback

The experiment included three sensory feedback conditions: no feedback (NF), auditory feedback (AF), and vibrotactile feedback (VF). In the NF condition, participants relied solely on visual information. In the AF condition, a 200 ms auditory tone (1,000 Hz) was delivered via a piezoelectric buzzer. In VF condition, a 200 ms vibration was generated by the stylus-mounted motor. In both feedback conditions, sensory input was triggered only when the stylus made contact with the first target, regardless of whether the trial involved one or two targets. The timing of feedback delivery was precisely controlled and synchronized with the moment of target contact.

Restricting augmented feedback to the first target was a theoretically driven decision. The One-Target Advantage (OTA) reflects the interaction between execution of the initial movement segment and preparation of the subsequent movement, making the first target a critical transition point between movement segments. Providing feedback at this point in the movement sequence allowed us to examine how enhanced sensory confirmation of first-segment completion influences preparation and transition into the next movement segment. In the present study, Pause Time (PT), defined as the duration of time spent at the first target, before initiation of the second movement segment, served as a key indicator of inter-segment processing. Two main hypotheses explain OTA (Adam et al., 2000; Helsen et al., 2001; Lavrysen et al., 2002; Khan et al., 2010; Lawrence et al., 2013, 2016; Bested et al., 2018; Hoffmann, 2017). According to the Movement Integration Hypothesis (MIH), planning of the second movement overlaps with execution of the first movement, resulting in shorter pauses and smoother transitions. In contrast, the Movement Constraint Hypothesis (MCH) proposes that the first movement segment is strategically controlled to optimize the second, potentially leading to longer dwell times at the intermediate target. Therefore, delivering feedback specifically at first-target contact allowed us to evaluate how augmented sensory information influenced movement transition processes, without directly interfering with continuous online control during ongoing movement execution.

### 2.4 Procedure

Participants completed a total of fifteen experimental blocks, created by combining five distinct movement sequences with three types of sensory feedback. Each block consisted of 15 trials of a specific task–feedback pairing, resulting in 225 trials across the entire session. To minimize potential order and learning effects, block presentation was systematically counterbalanced using a Latin square arrangement. At the beginning of each block, participants were informed about the upcoming movement sequence and feedback condition. Short rest intervals of approximately five minutes were provided between blocks to reduce fatigue and maintain performance consistency.

Prior to the experimental trials, participants performed a brief sensory detection task consisting of five trials for each feedback modality (AF, VF). During this task, a randomized go/no-go paradigm was used in which participants indicated detection of the stimulus by lifting the stylus.

At the start of each experimental trial participants positioned both hands-on predefined home locations to standardize initial posture. The right-hand stylus was placed on the right home square, while the left hand occupied its corresponding start position. This consistent setup ensured that comparisons across unimanual and bimanual conditions were not confounded by differences in starting configuration. This position triggered a randomized foreperiod ranging from 1,500 to 2,500 ms, after which the targets changed color to yellow to signal movement initiation. Participants were already aware of the required task for each block, so the visual stimulus functioned only as a timing trigger (i.e., go-signal).

**Figure 1:**
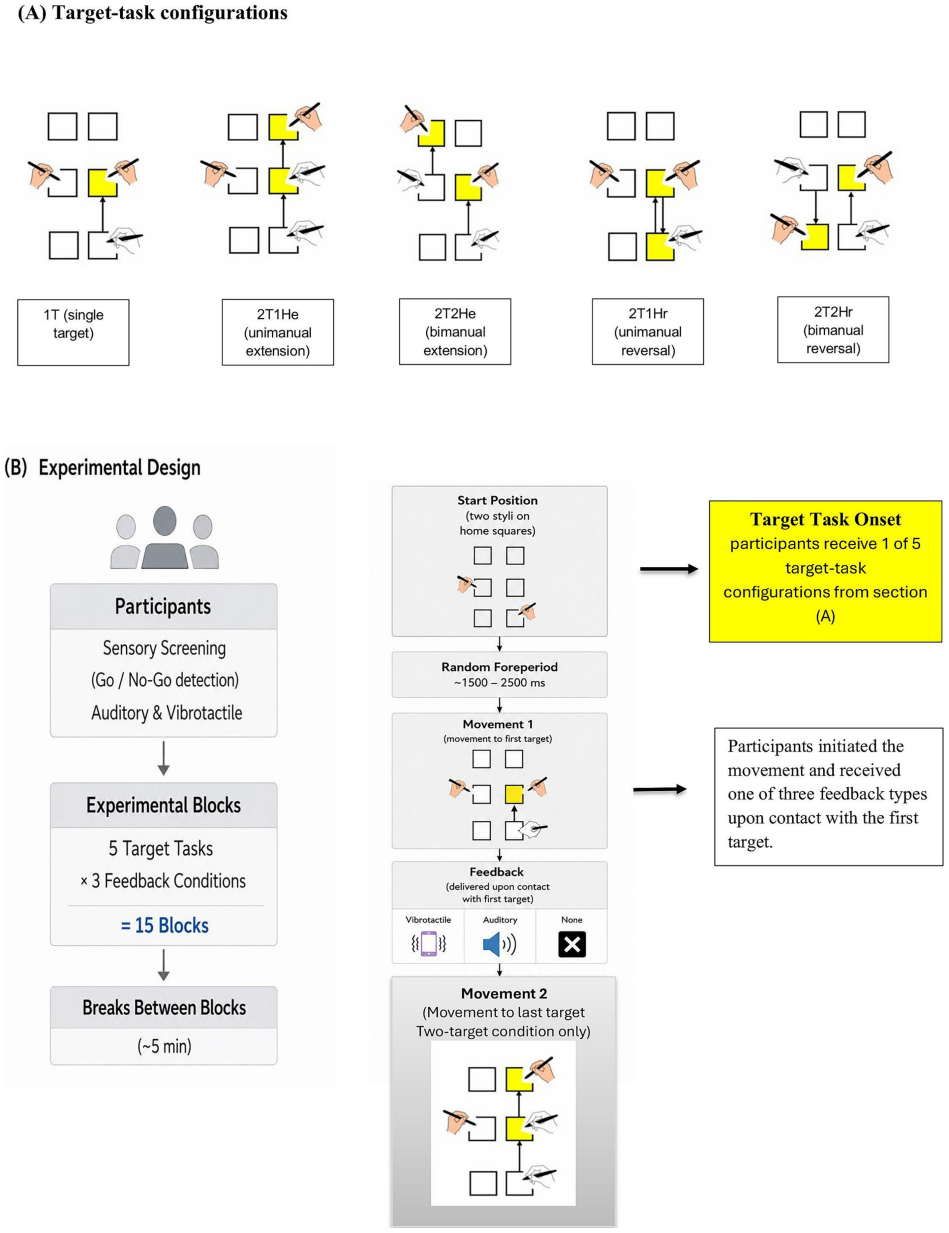
Experimental task and trial structure. **(A)** Target-task configurations: Participants performed five reaching conditions: (1T) single-target movement using the right hand; (2T1He) two-target unimanual extension (right hand moves from Target 1 to Target 2); (2T1Hr) two-target unimanual reversal (right hand returns from Target 1 to the home position); (2T2He) bimanual extension (right hand moves to Target 1 followed by left hand to Target 2); and (2T2Hr) bimanual reversal (right hand moves to Target 1 followed by left hand returning to the home position). **(B)** Trial sequence: Each trial began with both styli placed on home positions, followed by a variable foreperiod (1,500–2,500 ms). Upon target onset, participants executed the required movement as quickly and accurately as possible. Augmented sensory feedback (auditory or vibrotactile) was delivered only upon contact with the first target, regardless of task type.

Five reaching conditions are illustrated in Figure 1A and described below.

#### 2.4.1 Single-target task (1T)

Participants performed a single, rapid reaching movement with the right hand from the home position to Target 1. The left hand remained stationary.

#### 2.4.2 Unimanual extension task (2T1He)

Participants executed a two-segment movement using the right hand only, moving from the home position to Target 1 and then continuing forward to Target 2 in the same direction.

#### 2.4.3 Unimanual reversal task (2T1Hr)

Participants performed a two-segment movement with the right hand, first reaching Target 1 and then reversing direction back toward the home position.

#### 2.4.4 Bimanual extension task (2T2He)

Participants performed a sequential two-hand movement. The right hand first moved from the home position to Target 1, followed by the left hand extending from its home position (Target1) to Target 2.

#### 2.4.5 Bimanual reversal task (2T2Hr)

Participants again used both hands sequentially. The right hand first reached Target 1, after which the left hand performed a reversal movement from its home position (Target 1) back toward its home position.

Across all conditions, participants were instructed to prioritize both speed and accuracy, executing each movement segment as smoothly as possible while allowing natural corrective adjustments when necessary. For both two-hand conditions, participants were instructed to complete the first movement before initiating the second, while transitioning between movements as quickly as possible (Figures 1A,B).

### 2.5 Dependent measures

Dependent variables were selected to capture distinct components of sequential reaching control. Reaction time indexed movement preparation; pause time indexed transition processing between movement segments; movement time indexed execution duration; peak velocity and the timing of peak velocity described kinematic organization; and endpoint error measures assessed whether changes in timing or kinematics occurred at the cost of endpoint accuracy or consistency. Constant error was used to assess directional bias relative to the target, whereas variable error was used to assess trial-to-trial consistency of endpoint placement. Participants were instructed to move as quickly and accurately as possible; therefore, endpoint accuracy measures were included to evaluate possible speed–accuracy trade-offs. To improve clarity, dependent variables were grouped into conceptual domains reflecting movement preparation, movement transition, movement execution, kinematic control, and endpoint accuracy. Table 1 provides the abbreviation, full name, and interpretation of each dependent variable.

**Table 1:** Variable calculations and interpretations.

Domain	Variable	Full name	Variable calculation and interpretation
Preparation	RT	Reaction time	Time interval between the presentation of the go-signal and the initiation of movement. Movement onset is identified as the first data point at which resultant velocity exceeds 30 mm/s and remains above this threshold for at least 30 ms.
Execution	MT1	Movement time to the first target	Duration from movement onset to the termination of the first movement segment. The end of Segment 1 is defined as the first instance following Target 1 acquisition where resultant velocity drops below 30 mm/s for a minimum of 30 ms.
Execution	MT2	Movement time tothe second target	Time from the onset of the second movement segment to its completion at the final target. Segment 2 end is defined as the first sample where resultant velocity falls below 30 mm/s for at least 30 ms at the final target location.
Transition	PT	Pause time	Time elapsed between the completion of Segment 1 and the initiation of Segment 2. Specifically, this is measured from the first instance of sustained velocity below 30 mm/s (≥30 ms) at Target 1 to the first subsequent instance where velocity exceeds 30 mm/s for at least 30 ms.
Kinematic	PV1	Peak velocity during first segment movement	The highest resultant velocity recorded during the first movement segment (from movement onset to the end of Segment 1).
Kinematic	TTPV1	Time to peak velocity during first segment movement	Time interval between movement onset and the data point at which peak velocity (PV1) is reached within Segment 1.
Kinematic	TAPV1	Time after peak velocity during first segment movement	Duration from the occurrence of peak velocity (PV1) to the end of Segment 1, representing the deceleration phase of the movement.
Kinematic	PV2	Peak velocity during second segment movement	Maximum resultant velocity achieved during the second movement segment (from Segment 2 onset to Segment 2 completion).
Kinematic	TTPV2	Time to peak velocity during second segment movement	Time from the onset of Segment 2 to the point at which PV2 occurs.
Kinematic	TAPV2	Time after peak velocity during second segment movement	Time interval between the occurrence of PV2 and the end of Segment 2, reflecting deceleration toward the final target.
Accuracy	CE1	Constant error at the first target	Signed spatial deviation of the endpoint at Target 1 relative to the participant-specific calibrated target centre. Endpoint error was computed as the directional difference between the recorded stylus position at Segment 1 termination and the calibrated reference position, with positive and negative values indicating systematic overshoot or undershoot, respectively.
Accuracy	VE1	Variable error at first target	Intra-individual variability in endpoint accuracy at Target 1, quantified as the dispersion of endpoints around the participant’s mean endpoint location across trials. All endpoint positions were expressed relative to the calibrated target reference prior to variability estimation.
Accuracy	CE2	Constant error at second target	Signed spatial deviation of the final endpoint at Target 2 relative to the participant-specific calibrated target centre. This measure reflects systematic directional bias in final positioning, computed as the difference between the endpoint position at Segment 2 completion and the calibrated target location.
Accuracy	VE2	Variable error at second target	Trial-to-trial variability in final endpoint accuracy at Target 2, calculated as the spread of endpoint positions around the participant’s mean endpoint across trials. Endpoint coordinates were first aligned to the calibrated target centre to ensure consistency in spatial error estimation.

### 2.6 Data processing and analysis

Custom software developed in E-Prime 3.0 (Psychology Software Tools, Sharpsburg, PA, USA) was used to control stimulus presentation and deliver augmented feedback. The system also utilized a Chronos response and stimulus device (Model Chronos v1.4, Psychology Software Tools, Sharpsburg, PA, USA) to send TTL pulses to the motion capture system via a parallel port trigger. This ensured precise temporal alignment between visual feedback and movement recordings. After the experimental task was completed, participants performed a spatial target calibration.

Movement segmentation was performed from the stylus velocity signal using predefined amplitude and temporal criteria. The onset of movement was identified when velocity exceeded 30 mm/s for a continuous period of at least 30 ms, whereas movement termination was defined when velocity remained below this threshold for the same duration.

Participants held the stylus at the perceived centre of the target for 2 s to establish an individualized calibration reference for spatial error calculations. This recorded value was used for spatial error calculation. Position data were low-pass filtered using a dual-pass second-order Butterworth filter (cutoff: 15 Hz). Outliers were identified at the trial level within each task-feedback dataset. Trials were flagged if any primary dependent variable exceeded ±2.5 standard deviations (SD) from the condition-specific mean. Flagged trials were removed only after all dependent variables had been screened, so each trial was removed once even if it exceeded the threshold for multiple variables. Removed trials were saved separately with the reason for exclusion. Approximately 5% of trials were excluded before participant-level condition means were calculated. Assumptions of normality (Shapiro–Wilk), homogeneity (Levene’s test), and sphericity (Mauchly’s test) were verified prior to inferential analysis. Greenhouse–Geisser corrections were specified *a priori* for cases in which violations of sphericity were detected; however, no substantial violations requiring correction were observed in the reported analyses.

### 2.7 Outlier identification and exclusion

A ± 2.5 SD threshold was selected as a conservative criterion that identifies extreme atypical observations while retaining the vast majority of valid trials. Standard deviation (z-score)-based outlier exclusion has been shown through simulation studies to introduce relatively small bias compared with alternative exclusion methods, whereas retaining all outliers can substantially reduce statistical power and increase Type II error (Berger and Kiefer, 2021). Furthermore, recent methodological work emphasizes that the primary concern is not the specific SD threshold selected, but rather the *post hoc* selection among multiple outlier-removal methods after inspecting the data, which can inflate false-positive findings (Vankov, 2023). Accordingly, our ±2.5 SD criterion was specified a priori and applied consistently across all participants and experimental conditions, thereby avoiding analytical flexibility. Applying the criterion separately within each task-feedback condition also ensures that trial screening reflects the variability of each experimental condition rather than a pooled distribution. This approach is consistent with response-time analyses commonly used in motor control and motor learning research, where SD-based characterization of response variability is routinely employed (Ota et al., 2016). Following trial-level preprocessing and outlier removal, participant-level condition means were calculated for all dependent variables. All statistical analyses were conducted using SPSS version 28.0 (IBM Corp., Armonk, NY, USA). For first-segment variables, separate 3 (Feedback Condition) × 5 (Target Task) repeated-measures ANOVAs were conducted. For second-segment variables, 3 (Feedback Condition) × 4 (Target Task) repeated-measures ANOVAs were performed. Bonferroni-adjusted pairwise comparisons were used to follow up significant main effects and interactions. The significance level (*α*-level) was set at 0.05 for the omnibus and post-hoc tests.

## 3 Results

Significant results are reported below; a complete summary of findings is presented in Supplementary Tables S1–S3. Dependent variables were organized into conceptual domains reflecting movement preparation, transition, execution, kinematic control, and endpoint accuracy. Estimated marginal means for all conditions across the first and second movement segments are also provided in the Supplementary Tables S4, S5.

### 3.1 Movement preparation

#### 3.1.1 Reaction time (RT)

**Figure 2:**
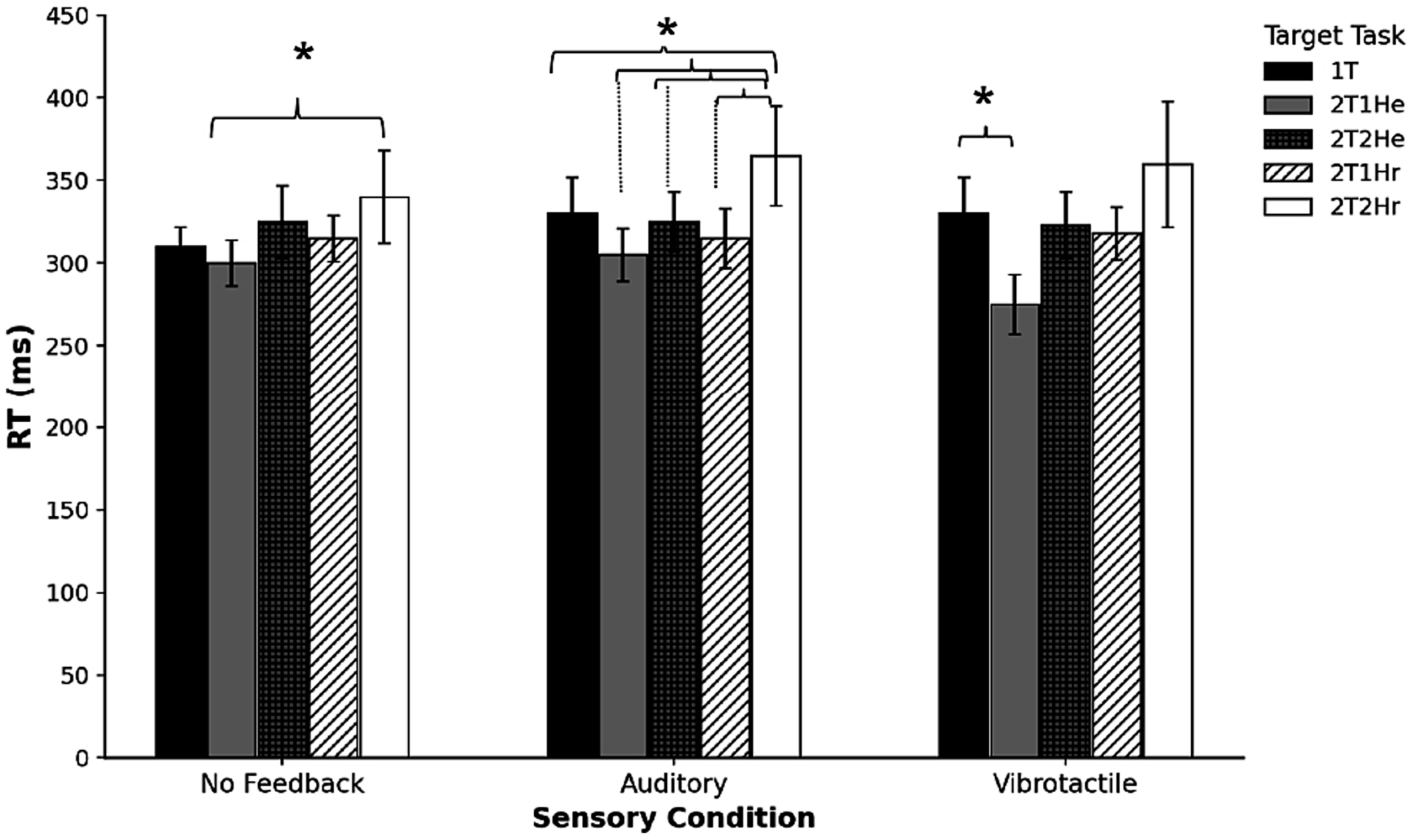
Reaction time across feedback conditions and target tasks. Mean reaction time (RT ± SE) for the single-target task (1 T), unimanual extension task (2T1He), bimanual extension task (2T2He), unimanual reversal task (2T1Hr), and bimanual reversal task (2T2Hr) under no-feedback, auditory feedback, and vibrotactile feedback conditions. Significant pairwise comparisons are indicated by brackets and asterisks (*p < 0.05). Under no feedback, significant differences were observed between the unimanual extension and bimanual reversal tasks. Under auditory feedback, the bimanual reversal task differed significantly from all other task conditions. Under vibrotactile feedback, significant differences were observed between the single-target and unimanual extension tasks, and between the single-target and bimanual reversal tasks.

RT differed significantly across target tasks, *F*(4, 92) = 10.83, *p* < 0.001, ηp^2^ = 0.32. *Post hoc* comparisons showed that reaction time was shorter in the unimanual extension task (M = 289 ms, SE = 7 ms) than in the single-target, bimanual extension, and bimanual reversal tasks. The longest reaction times were observed in the bimanual reversal task. As shown in Figure 2, a significant interaction was also observed between feedback condition and target task, *F*(8, 184) = 2.92, *p* = 0.004, ηp^2^ = 0.11. Under no feedback, reaction time was slower in the bimanual reversal task than in the unimanual extension task (*p* = 0.02). In the auditory condition, the bimanual reversal task produced longer reaction times than all other tasks (all ps ≤ 0.01). Under vibrotactile feedback, reaction time in the unimanual extension task was faster than in the single-target task (*p* < 0.001), whereas the bimanual reversal task remained significantly slower than the single-target task (*p* < 0.001).

### 3.2 Movement execution

#### 3.2.1 Movement time to the first target (MT1)

No significant main effects of feedback condition, *F*(2, 46) = 0.10, *p* = 0.902, ηp^2^ = 0.004, or target task, *F*(4, 92) = 0.62, *p* = 0.652, ηp^2^ = 0.03, were observed for first-segment movement time (MT1). The interaction between feedback condition and target task was also not significant, *F*(8, 184) = 0.78, *p* = 0.619, ηp^2^ = 0.03.

#### 3.2.2 Movement time to the second target (MT2)

A significant main effect of feedback condition was observed for second-segment movement time (MT2), *F*(2, 46) = 3.42, *p* = 0.041, ηp^2^ = 0.13. Vibrotactile feedback produced the shortest MT2, followed by auditory feedback and no feedback, although Bonferroni-adjusted pairwise comparisons were not significant.

A significant main effect of target task was also found, *F*(3, 69) = 23.18, *p* < 0.001, ηp^2^ = 0.50. *Post hoc* comparisons showed that MT2 was shortest during the unimanual reversal task and significantly longer during the unimanual extension, bimanual extension, and bimanual reversal tasks. The longest MT2 values were observed in bimanual conditions. Importantly, a significant interaction emerged between feedback condition and target task, *F*(6, 138) = 6.47, *p* < 0.001, ηp^2^ = 0.22, indicating that the effects of feedback varied across task demands. Follow-up post-hoc comparisons revealed that vibrotactile feedback significantly reduced MT2 during the bimanual extension task relative to both no-feedback and auditory conditions (ps < 0.05). In contrast, no feedback-related differences were observed in the simpler unimanual tasks. Overall, vibrotactile feedback selectively improved performance under higher coordination demands.

### 3.3 Movement transition

#### 3.3.1 Pause time (PT)

Pause time differed significantly across target tasks, *F*(3, 69) = 334.14, *p* < 0.001, ηp^2^ = 0.93. *Post hoc* comparisons showed significant differences between all task conditions (all ps < 0.001). The longest pause times were observed during the bimanual extension task, whereas the shortest pause times occurred during the unimanual reversal task. In addition, both bimanual tasks produced longer pause times than the unimanual tasks, indicating greater transition demands during interlimb coordination.

### 3.4 Endpoint accuracy

No significant main effects or interactions were observed for endpoint accuracy and consistency measures (CE1, VE1, CE2, VE2) across feedback conditions or target task complexity (all ps > 0.05). These suggest that participants were able to maintain consistent endpoint control regardless of feedback type or movement complexity.

### 3.5 Kinematic control

#### 3.5.1 Peak velocity during the second movement segment (PV2)

Peak velocity during the second movement segment differed significantly across feedback conditions, *F*(2, 46) = 17.02, *p* < 0.001, ηp^2^ = 0.43. Pairwise comparisons showed that vibrotactile feedback produced significantly higher PV2 than both the no-feedback condition (*p* < 0.001) and the auditory condition (*p* < 0.001). No significant difference was observed between no-feedback and auditory feedback (*p* = 1.00).

A significant main effect of target task was also found, *F*(3, 69) = 6.29, *p* < 0.001, ηp^2^ = 0.22. *Post hoc* comparisons revealed that PV2 was highest during the unimanual reversal task, which was significantly greater than during the unimanual extension and bimanual extension tasks. No other pairwise differences were significant after correction.

Importantly, a significant interaction emerged between feedback condition and target task, *F*(6, 138) = 11.59, *p* < 0.001, ηp^2^ = 0.34, indicating that the effect of feedback varied across task demands. Follow-up comparisons showed that vibrotactile feedback produced substantially higher PV2 during the unimanual extension and bimanual extension tasks compared to both no-feedback and auditory conditions (ps < 0.01). In contrast, sensory-related differences were reduced or absent during the reversal tasks.

#### 3.5.2 Time to peak velocity during the second movement segment (TTPV2)

No significant main effect of feedback condition was observed for time to peak velocity during the second movement segment (TTPV2), *F*(2, 46) = 2.71, *p* = 0.077, ηp^2^ = 0.11. Pairwise comparisons further confirmed that TTPV2 did not differ significantly across no-feedback, auditory, and vibrotactile conditions (all ps > 0.13).

In contrast, a significant main effect of target task was found, *F*(3, 69) = 19.87, *p* < 0.001, ηp^2^ = 0.46, indicating that TTPV2 increased with task complexity. *Post hoc* comparisons showed that the shortest TTPV2 occurred during the unimanual extension task, whereas the longest TTPV2 was observed during the bimanual reversal task, which was significantly longer than all other tasks (all ps < 0.015).

**Figure 3:**
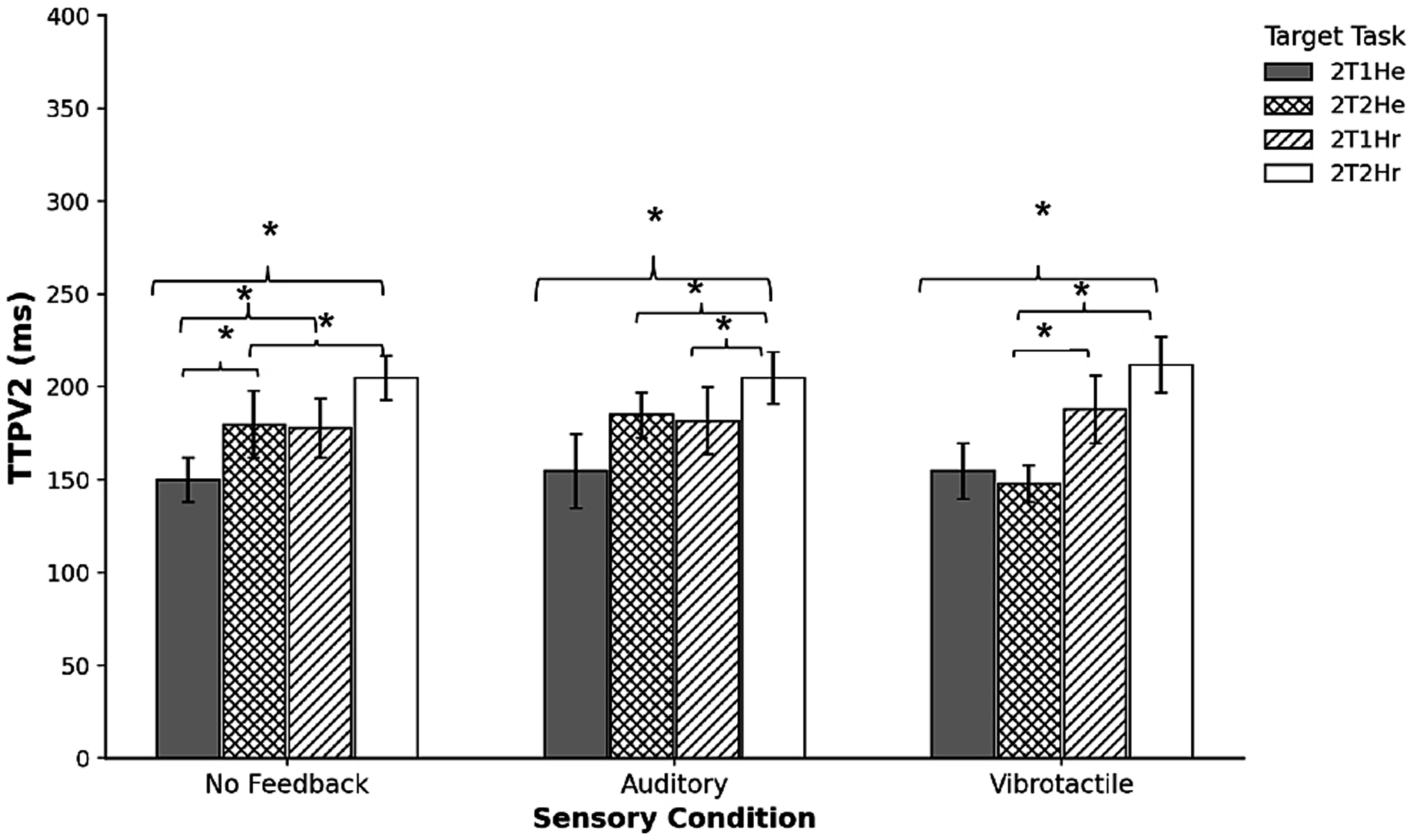
Time to peak velocity during the second movement segment (TTPV2). Mean time to peak velocity during the second movement segment (TTPV2 ± SE) across feedback conditions (no-feedback, auditory feedback, vibrotactile feedback) and two-target task configurations, including unimanual extension (2T1He), bimanual extension (2T2He), unimanual reversal (2T1Hr), and bimanual reversal (2T2Hr). Significant pairwise comparisons are indicated by brackets and asterisks (*p < 0.05). Significant differences were observed primarily between the unimanual extension and bimanual reversal tasks across feedback conditions. Under vibrotactile feedback, the bimanual extension task also differed significantly from the bimanual reversal task.

As shown in Figure 3, a significant interaction also emerged between feedback condition and target task, *F*(6, 138) = 8.47, *p* < 0.001, ηp^2^ = 0.27, indicating that the effect of feedback varied across movement tasks. Under no feedback condition, TTPV2 increased progressively from the simpler extension tasks to the more complex reversal tasks. Vibrotactile feedback selectively reduced TTPV2 during the bimanual extension task relative to both no-feedback and auditory conditions (ps < 0.02). In contrast, no feedback-related differences were observed in the simplest unimanual extension task, and effects were less consistent during reversal tasks.

#### 3.5.3 Time after peak velocity during the second movement segment (TAPV2)

A significant main effect of feedback condition was observed for time after peak velocity during the second movement segment (TAPV2), *F*(2, 46) = 7.21, *p* = 0.002, ηp^2^ = 0.24. *Post hoc* comparisons showed that TAPV2 was significantly longer in the no-feedback condition than in the vibrotactile condition. No other pairwise comparisons reached significance, although the difference between no-feedback and auditory feedback approached significance (*p* = 0.055).

A significant main effect of target task was also found, *F*(3, 69) = 31.29, *p* < 0.001, ηp^2^ = 0.58, indicating clear differences across movement tasks. The longest TAPV2 values were observed during the bimanual extension and bimanual reversal tasks, whereas the shortest TAPV2 occurred during the unimanual reversal task. Post hoc comparisons confirmed that TAPV2 in the unimanual reversal task was significantly shorter than in all other task conditions (all ps < 0.001).

**Figure 4:**
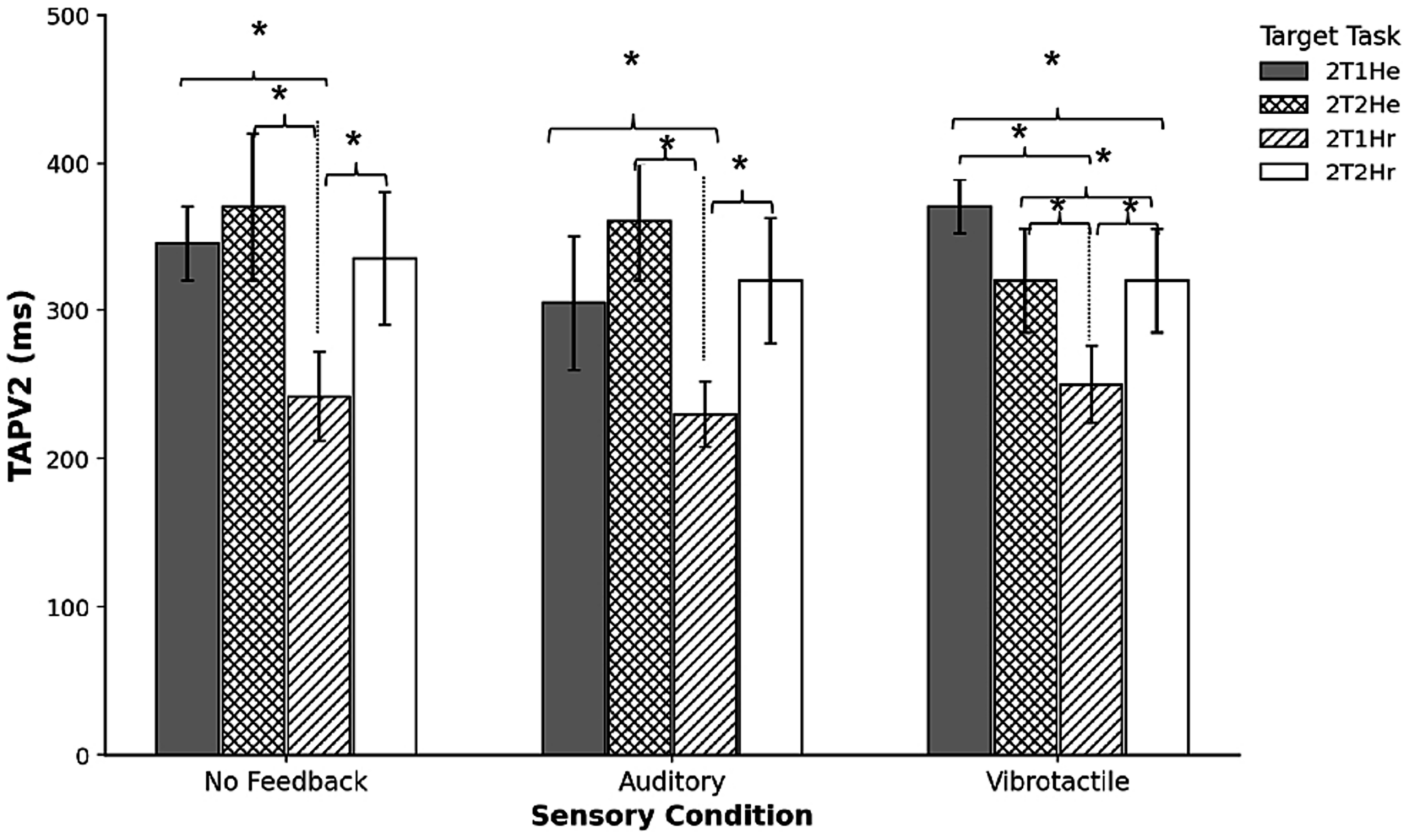
Time after peak velocity during the second movement segment (TAPV2). Mean time after peak velocity during the second movement segment (TAPV2 ± SE) across feedback conditions (no-feedback, auditory feedback, vibrotactile feedback) and two-target task configurations, including unimanual extension (2T1He), bimanual extension (2T2He), unimanual reversal (2T1Hr), and bimanual reversal (2T2Hr). Significant pairwise comparisons are indicated by brackets and asterisks (*p < 0.05). Under no-feedback and auditory conditions, significant differences were observed between the unimanual reversal task and both extension tasks. Under vibrotactile feedback, significant differences were observed between the unimanual reversal and bimanual reversal tasks, as well as between the bimanual extension and unimanual reversal tasks.

As shown in Figure 4, a significant interaction emerged between feedback condition and target task, *F*(6, 138) = 3.83, *p* = 0.001, ηp^2^ = 0.14, indicating that the influence of feedback varied across task demands. Under no feedback, TAPV2 was significantly longer during the unimanual extension and bimanual extension tasks compared to the unimanual reversal task (ps < 0.001). A similar pattern was observed in the auditory condition, where the unimanual reversal task again produced shorter TAPV2 values than the other tasks (ps < 0.02). Under vibrotactile feedback, TAPV2 remained shortest during the unimanual reversal task, whereas the bimanual reversal task produced significantly longer TAPV2 values (ps < 0.01).

## 4 Discussion

The present study examined how augmented sensory feedback influences sequential reaching performance in older adults under varying levels of task complexity. By extending prior work from simple one- and two-target tasks to more complex sequences involving reversal and bimanual coordination, the findings clarify how task demands and sensory feedback influence motor preparation and online control in older adults. When considered alongside our previous studies involving younger adults (Mohammadalinezhad Kolahdouz et al., 2026b) and direct age-group comparisons (Mohammadalinezhad Kolahdouz et al., 2026a), the results suggest that aging may modify the expression of these task and feedback-related effects. Overall, vibrotactile feedback produced task-dependent benefits, most evident in reaction time and second-segment kinematics, with minimal effects on first-segment execution or endpoint accuracy.

A primary finding was the significant interaction between feedback condition and target task for reaction time (Figure 2), in which *post hoc* comparisons revealed that vibrotactile feedback significantly reduced RT, specifically in the unimanual extension task relative to the one-target task. This finding demonstrates that the traditional one-target advantage (OTA) was effectively reversed in this context. The reduction in reaction time is intriguing because feedback was not delivered until first-target contact, and therefore cannot be attributed to a facilitation of online corrections during movement execution itself. Rather, one possible interpretation of the RT results is that participants used the expected sensory consequence to facilitate their movement preparation. Adjustments to movement preparation processes were possible in the present task because participants knew that a consistent vibrotactile signal would occur at the first target, which may have reduced uncertainty regarding segment transitions and facilitated advance organization of the sequential action.

This interpretation is further supported by the observation that feedback effects emerged primarily in reaction time, while movement time to the first segment of the task and endpoint accuracy remained largely unaffected, suggesting that sensory feedback influenced preparatory processes more than early execution control. Importantly, the augmented feedback used in the present study did not provide explicit information about movement accuracy or task success. Instead, the auditory and vibrotactile cues served as temporally precise sensory events signaling first-target contact. Vibrotactile feedback may therefore have supplemented somatosensory information available during movement transitions (Goodman and Tremblay, 2021; Risi et al., 2019). In addition, augmented sensory cues may have influenced attentional allocation and expectancy during movement preparation. Anticipation of sensory feedback has been shown to engage preparatory sensorimotor processes prior to movement initiation and may direct attention toward movement-relevant events (Wulf and Lewthwaite, 2016). In the present study, vibrotactile feedback may have reinforced intrinsic proprioceptive confirmation of first-target acquisition, whereas auditory feedback may have primarily functioned as an external temporal cue supporting movement timing. In addition to supplementing somatosensory information, augmented feedback may also have influenced attentional allocation during movement preparation and transition phases. Augmented sensory feedback can increase the salience of task-relevant information and direct attention toward critical movement events, potentially facilitating movement organization and online monitoring processes (Sigrist et al., 2013; Robak et al., 2026). In the present study, vibrotactile feedback may have enhanced intrinsic proprioceptive processing by reinforcing sensory confirmation of first-target acquisition, whereas auditory feedback may have primarily acted as an external temporal cue directing attention toward movement timing rather than limb-specific regulation (Risi et al., 2019; Sigrist et al., 2013; Pomplun et al., 2022). This interpretation aligns with evidence that older adults rely heavily on somatosensory input during planning (Goodman and Tremblay, 2021) and with previous findings showing that vibrotactile feedback can alter OTA-related temporal costs (Mohammadalinezhad Kolahdouz et al., 2026a).

Importantly, this reversal of the OTA was task specific. The results showed that the facilitation emerged only in the unimanual extension task and did not extend to unimanual reversal task, bimanual reversal task, or bimanual extension tasks. In simple extension sequences, spatial alignment between targets may facilitate overlap between planning and execution, whereas reversal and limb-switch tasks impose additional constraints by requiring directional or effector reorganization. Consistent with prior work (Lavrysen et al., 2002; Lawrence et al., 2016), these findings suggest that vibrotactile feedback may have enhanced preparatory processes specifically when the movement sequence supported integrated planning. Previous research has shown that in sequential movements involving the same effector and a continuous spatial trajectory, performers can partially prepare subsequent movement elements before the first segment is completed, resulting in greater temporal overlap between planning and execution processes. In the present study, the unimanual extension task involved a forward extension movement using the same limb across both targets, which may have facilitated advance organization of the second segment during preparation of the first. In contrast, reversal and bimanual conditions required directional changes or switching between effectors, increasing coordination and reorganization demands and reducing the extent to which movement segments could be integrated in advance.

Under these more complex conditions, the benefits of vibrotactile feedback on movement initiation were no longer observed. In contrast, first-segment movement time was unaffected by feedback or task. This suggests that once initiated, the initial reach was governed largely by feedforward control and remained stable across conditions. This differs from our previous findings using simpler task conditions (Mohammadalinezhad Kolahdouz et al., 2026a), indicating that feedback-related effects on first-segment movement time may diminish as task complexity increases. By comparison, second-segment movement time was strongly influenced by task complexity. The longest durations occurred in bimanual extension and reversal conditions, whereas shorter MT2 values were observed in single-hand tasks. This pattern reflects increased coordination and planning demands when switching effectors or maintaining more complex sequences, consistent with evidence that aging disproportionately affects multi-step actions (Sarlegna, 2006; Wang et al., 2020; Zhang et al., 2024). Moreover, similar task-related effects have been reported in younger adults performing comparable sequential reaching tasks (Mohammadalinezhad Kolahdouz et al., 2026b), suggesting that these effects primarily reflect increasing task complexity. However, previous age-group comparisons indicate that such demands may place greater burdens on sensorimotor control in older adults (Mohammadalinezhad Kolahdouz et al., 2026a). Longer pause times in bimanual conditions further suggest greater difficulty during movement transitions under higher coordination demands. Notably, vibrotactile feedback selectively reduced MT2 in the demanding bimanual extension task. Unlike the reaction time effect observed in simpler tasks, this benefit emerged under high coordination demands, suggesting that vibrotactile feedback supports different stages of control depending on task complexity facilitating preparation in simple sequences and execution in more complex ones. This extends previous findings in younger adults, where vibrotactile feedback selectively enhanced late-phase control rather than globally reducing movement time (Mohammadalinezhad Kolahdouz et al., 2026b). In contrast, auditory feedback produced comparatively limited effects across most outcome measures. This pattern is consistent with previous work suggesting that auditory cues primarily provide information about movement timing, whereas vibrotactile feedback may provide more movement-specific somatosensory information that can support online movement regulation and sequential control (Sigrist et al., 2013; Bark et al., 2014).

Kinematic analyses further revealed that vibrotactile feedback increased peak velocity during the second movement segment and reduced time after peak velocity during this second segment movement, with task-specific reductions in time to peak velocity during the second movement segment. These changes indicate more efficient movement organization rather than uniform speeding. Increased peak velocity during second segment movement reflects a stronger movement impulse, whereas reduced TAPV2 indicates improved terminal control. Consistent with the Multiple Process Model (Elliott et al., 2010, 2017), which proposes that goal-directed movements involve interacting processes of movement planning, impulse control, and late limb-target regulation, these findings suggest that vibrotactile feedback primarily enhanced late-phase limb-target control rather than early preparatory processes. Specifically, the increase in peak velocity during second movement segment, together with the reduction in time after peak velocity during the second movement segment, indicates more efficient online regulation during the deceleration and target-approach phase of the movement. According to the model, late limb-target control relies on sensory feedback to reduce discrepancies between the limb and target position as the movement nears completion, whereas impulse control reflects earlier online adjustments based on expected sensory consequences of the movement. The above pattern replicates prior findings showing improved late-phase movement regulation during deceleration without measurable changes in endpoint accuracy (Mohammadalinezhad kolahdouz et al., 2026b).

Task-related differences in time to peak velocity during the second movement segment, and peak velocity during the second movement segment, also support adaptive control strategies in older adults. Longer time to peak velocity during the second movement segment in complex conditions may reflect a more cautious early movement phase, consistent with evidence that older adults rely on increased planning and online control when task demands increase (Elliott et al., 2010; Seidler et al., 2010). In contrast, higher peak velocity during the second movement segment indicates older adults made compensatory adjustments once the second segment was specified. These adjustments reflect their preserved ability to scale movement execution despite delayed initiation. Similar patterns have been interpreted as adaptive sensorimotor reorganization rather than generalized motor slowing in aging populations (Ketcham and Stelmach, 2004). Vibrotactile feedback may further support this transition by providing an additional sensory event at the first target, potentially enhancing movement updating and segment transition processes.

Despite these temporal and kinematic effects, endpoint accuracy remained stable across all conditions. No significant effects were observed for constant error or variable error measures, indicating that improvements in movement dynamics did not compromise either endpoint accuracy or endpoint consistency. This finding aligns with previous work demonstrating that older adults often prioritize accuracy during complex aiming tasks, even when movement strategies are modified (Elliott et al., 2001). The spatial accuracy findings are also consistent with studies showing that augmented sensory feedback can facilitate movement regulation without negatively affecting spatial accuracy (Mohammadalinezhad Kolahdouz et al., 2026a).

Taken together, these findings indicate that sequential movement organization in older adults is particularly affected by increased task complexity, with effects most evident in second-segment timing and control. Vibrotactile feedback does not uniformly enhance performance, but does provide selective, functionally meaningful benefits, improving preparation in simple sequences, and execution efficiency under higher demands. Specifically, vibrotactile feedback supported specific phases of sequential control, particularly movement segment transition and late-phase limb-target regulation.

## 5 Conclusion

This study demonstrates that vibrotactile feedback can selectively enhance sequential reaching performance in older adults, but that its effects depend strongly on task structure. Vibrotactile feedback reduced reaction time in the single-hand extension task, reversing the typical OTA pattern, and improved second-segment execution by increasing peak velocity and reducing deceleration time, particularly under higher coordination demands. At the same time, first-segment movement time and endpoint accuracy remained stable, suggesting that augmented feedback primarily supported preparation and online control rather than producing global changes in movement speed or end point accuracy and consistency. These findings extend our previous work by showing that feedback effects observed in simpler unimanual paradigms do not generalize uniformly to more complex actions but instead become increasingly task specific as reversal and bimanual coordination demands are introduced. More broadly, the results highlight the importance of considering both movement phase and task complexity when evaluating sensory augmentation in aging. Vibrotactile feedback appears to be most effective when it supports either the anticipatory organization of simple sequences or the online regulation of more demanding second-segment actions. This makes it a promising candidate for interventions designed to improve sequential motor performance in older adults without compromising endpoint accuracy.

## Data Availability

The raw data supporting the conclusions of this article will be made available by the authors upon request.
