# Supplementary material for: Sequential reaching in older adults: vibrotactile feedback improves preparation and movement control
Source: Front Aging Neurosci. 2026 Jul 17;18:1840948. doi: 10.3389/fnagi.2026.1840948 (PMC13424289; doi:10.3389/fnagi.2026.1840948)
Supplement: Supplementary file 1 [file Data_Sheet_1.PDF]

Table 1s. Repeated-measures ANOVA results for temporal performance measures across feedback conditions and target tasks.

Source	RT	MT1	MT2	Pause Time (PT)
Feedback Condition	$F(2,46)=2.65$, $p=.08$, $\eta p^2=.10$	$F(2,46)=0.10$, $p=.90$, $\eta p^2=.004$	$F(2,46)=2.418$, $p=.041$*, $\eta p^2=.129$	$F(2,46)=2.98$, $p=.743$, $\eta p^2=.01$
Target Task	$F(4,92)=10.83$, $p=.001$*, $\eta p^2=.32$	$F(4,92)=0.62$, $p=.65$, $\eta p^2=.03$	$F(3,69)=23.17$, $p<.001$*, $\eta p^2=.502$	$F(3,69)=334.14$, $p<.001$*, $\eta p^2=.94$
Feedback × Task	$F(8,184)=2.92$, $p=.004$*, $\eta p^2=.11$	$F(8,184)=0.78$, $p=.62$, $\eta p^2=.03$	$F(6,138)=6.470$, $p<.001$, $\eta p^2=.220$	$F(6,138)=0.79$, $p=.579$, $\eta p^2=.03$

Note. Temporal measures include reaction time (RT), movement time to the first target (MT1), movement time to the second target (MT2), and pause time at the first target (PT). F statistics, p values, and partial eta-squared effect sizes (ηp^2) are reported for the main effects of sensory condition and target task, as well as their interaction. * indicates statistically significant effects at $p \leq .05$.

Table 2s. Repeated-measures ANOVA results for endpoint accuracy measures across feedback conditions and target tasks.

Source	CE1	VE1	CE2	VE2
Feedback Condition	$F(2,46)=0.55$, $p=.580$, $\eta p^2=.023$	$F(2,46)=2.82$, $p=.070$, $\eta p^2=.11$	$F(2,46)=.044$, $p=.957$, $\eta p^2=.002$	$F(2,46)=1.13$, $p=.33$, $\eta p^2=.047$
Target Task	$F(4,92)=1.61$, $p=.178$, $\eta p^2=.07$	$F(4,92)=1.31$, $p=.273$, $\eta p^2=.05$	$F(3,69)=2.45$, $p=.071$, $\eta p^2=.096$	$F(3,69)=1.68$, $p=.178$, $\eta p^2=.07$
Feedback × Task	$F(8,184)=0.95$, $p=.474$, $\eta p^2=.04$	$F(8,184)=1.14$, $p=.337$, $\eta p^2=.05$	$F(6,138)=1.137$, $p=.344$, $\eta p^2=.047$	$F(6,138)=1.44$, $p=.203$, $\eta p^2=.06$

Note. Endpoint accuracy measures include constant error (CE) and variable error (VE) for the first (CE1, VE1) and second (CE2, VE2) movement segments. F statistics, p values, and partial eta-squared effect sizes (ηp^2) are reported for the main effects of sensory condition and target task, and their interaction. * indicates statistically significant effects at $p \leq .05$.

Table 3s. Repeated-measures ANOVA results for kinematic measures across feedback conditions and target tasks.

Source	PV1	PV2	TTPV1	TTPV2	TAPV1	TAPV2
Feedback Condition	$F(2,46)=0.03$, $p=.972$, $\eta p^2=.001$	$F(2,46)=17.02$, $p<.001$*, $\eta p^2=.425$	$F(2,46)=1.01$, $p=.373$, $\eta p^2=.04$	$F(2,46)=2.72$, $p=.077$, $\eta p^2=.106$	$F(2,46)=0.21$, $p=.812$, $\eta p^2=.01$	$F(2,46)=7.213$, $p=.002$*, $\eta p^2=.239$
Target Task	$F(4,92)=2.19$, $p=.077$, $\eta p^2=.09$	$F(3,69)=6.28$, $p<.001$*, $\eta p^2=.215$	$F(4,92)=0.79$, $p=.533$, $\eta p^2=.03$	$F(3,69)=19.873$, $p<.001$*, $\eta p^2=.464$	$F(4,92)=0.90$, $p=.465$, $\eta p^2=.04$	$F(3,69)=31.28$, $p<.001$*, $\eta p^2=.576$
Feedback × Task	$F(8,184)=1.64$, $p=.117$, $\eta p^2=.07$	$F(6,138)=11.59$, $p<.001$*, $\eta p^2=.335$	$F(8,184)=1.34$, $p=.228$, $\eta p^2=.06$	$F(6,138)=8.47$, $p<.001$*, $\eta p^2=.269$	$F(8,184)=0.92$, $p=.504$, $\eta p^2=.04$	$F(6,138)=3.830$, $p<.001$*, $\eta p^2=.143$

Note. Kinematic variables include peak velocity (PV), time to peak velocity (TTPV), and time after peak velocity (TAPV) during the first and second segments movement. F statistics, p values, and partial eta-squared effect sizes (ηp^2) are reported for the main effects of sensory condition and target task, and their interaction. * indicates statistically significant effects at $p \leq .05$.
