# Supplementary material for: Sequential reaching in older adults: vibrotactile feedback improves preparation and movement control
Source: Front Aging Neurosci. 2026 Jul 17;18:1840948. doi: 10.3389/fnagi.2026.1840948 (PMC13424289; doi:10.3389/fnagi.2026.1840948)
Supplement: Supplementary file 2 [file Data_Sheet_2.PDF]

Table 4s. Estimated Marginal Means (Mean ± SE) for First-Segment Variables

Feedback Condition	Target Task	RT (ms)	MT1 (ms)	CE1 (mm)	VE1 (mm)	PV1 (mm/s^2)	TTPV1 (ms)	TAPV1 (ms)
NF	1T	303.78 ± 7.34	457.50 ± 28.69	-0.734 ± 0.357	0.879 ± 0.193	766.80 ± 28.15	146.86 ± 6.35	310.64 ± 23.96
NF	2T1He	297.09 ± 7.07	453.16 ± 16.49	-0.293 ± 0.320	0.658 ± 0.127	739.00 ± 19.61	150.14 ± 8.95	303.02 ± 14.01
NF	2T2He	323.06 ± 10.27	453.64 ± 22.53	-0.461 ± 0.262	0.486 ± 0.035	719.08 ± 22.90	150.55 ± 5.78	303.09 ± 18.41
NF	2T1Hr	310.41 ± 9.31	454.38 ± 26.67	-0.410 ± 0.302	0.656 ± 0.164	748.75 ± 25.61	146.82 ± 6.18	307.57 ± 24.21
NF	2T2Hr	338.37 ± 14.70	444.96 ± 24.93	-0.503 ± 0.304	0.628 ± 0.098	759.83 ± 28.26	144.02 ± 5.55	300.94 ± 21.44
AF	1T	325.27 ± 12.41	465.64 ± 27.04	-0.768 ± 0.369	0.743 ± 0.164	753.86 ± 25.13	142.56 ± 5.33	323.09 ± 24.12
AF	2T1He	301.56 ± 9.00	454.10 ± 23.41	-0.398 ± 0.307	0.625 ± 0.124	734.73 ± 21.96	140.19 ± 4.78	313.91 ± 20.55
AF	2T2He	320.47 ± 10.43	455.11 ± 24.19	-0.196 ± 0.293	0.581 ± 0.071	738.71 ± 26.04	148.79 ± 5.53	306.32 ± 21.84
AF	2T1Hr	309.68 ± 10.18	453.29 ± 32.10	-0.712 ± 0.338	0.909 ± 0.232	741.23 ± 25.47	155.66 ± 9.56	297.63 ± 26.51
AF	2T2Hr	362.44 ± 16.39	438.91 ± 22.12	-0.382 ± 0.362	0.572 ± 0.080	771.25 ± 26.68	139.55 ± 4.91	299.36 ± 19.70
VF	1T	325.47 ± 10.46	461.37 ± 26.12	-1.107 ± 0.304	0.631 ± 0.109	752.73 ± 24.36	145.12 ± 6.44	316.26 ± 22.67
VF	2T1He	269.35 ± 10.31	468.00 ± 24.33	-0.394 ± 0.308	0.439 ± 0.042	723.52 ± 21.77	145.99 ± 5.18	322.02 ± 21.84

Feedback Condition	Target Task	RT (ms)	MT1 (ms)	CE1 (mm)	VE1 (mm)	PV1 (mm/s^2)	TTPV1 (ms)	TAPV1 (ms)
VF	2T2He	320.18 ± 13.12	435.65 ± 14.34	-0.382 ± 0.280	0.535 ± 0.055	753.26 ± 22.75	145.48 ± 4.96	290.16 ± 12.28
VF	2T1Hr	308.14 ± 10.64	439.06 ± 29.76	-0.358 ± 0.294	0.491 ± 0.077	755.94 ± 27.53	144.00 ± 6.45	295.06 ± 24.50
VF	2T2Hr	355.69 ± 19.39	452.29 ± 23.27	-0.570 ± 0.344	0.591 ± 0.098	749.78 ± 24.68	144.29 ± 4.72	307.99 ± 21.02

Note. NF = no-feedback condition; AF = auditory feedback condition; VF = vibrotactile feedback condition. 1T = single-target task; 2T1He = unimanual extension task; 2T2He = bimanual extension task; 2T1Hr = unimanual reversal task; 2T2Hr = bimanual reversal task. Values represent estimated marginal means ± standard error (SE).

Table 5s. Estimated Marginal Means (Mean ± SE) for Second-Segment Variables

Feedback Condition	Target Task	MT2 (ms)	PT (ms)	CE2 (mm)	VE2 (mm)	PV2 (mm/s^2)	TTPV2 (ms)	TAPV2 (ms)
NF	2T1He	451.37 ± 15.28	114.18 ± 13.30	-0.465 ± 0.258	0.540 ± 0.069	639.85 ± 21.91	150.45 ± 7.69	346.75 ± 12.99
NF	2T2He	533.91 ± 25.97	79.70 ± 10.67	-0.868 ± 0.409	0.603 ± 0.085	606.74 ± 19.56	177.31 ± 7.63	370.88 ± 24.91
NF	2T1Hr	415.61 ± 21.00	840.25 ± 55.22	0.241 ± 0.310	0.756 ± 0.128	730.70 ± 27.21	176.07 ± 6.97	239.55 ± 17.43
NF	2T2Hr	532.93 ± 28.12	957.43 ± 45.25	0.102 ± 0.396	0.926 ± 0.158	690.47 ± 24.29	201.67 ± 6.92	331.25 ± 23.29
AF	2T1He	459.85 ± 22.14	141.15 ± 17.20	-0.828 ± 0.359	1.107 ± 0.301	651.30 ± 26.77	155.64 ± 9.40	304.22 ± 21.18
AF	2T2He	540.51 ± 28.15	64.13 ± 9.59	-0.582 ± 0.462	0.666 ± 0.094	601.30 ± 23.83	183.19 ± 8.33	357.32 ± 23.59

**Feedback Condition**	**Target Task**	**MT2 (ms)**	**PT (ms)**	**CE2 (mm)**	**VE2 (mm)**	**PV2 (mm/s^2)**	**TTPV2 (ms)**	**TAPV2 (ms)**
AF	2T1Hr	406.97 ± 15.08	800.76 ± 43.65	-0.176 ± 0.407	0.956 ± 0.254	708.09 ± 33.00	180.41 ± 7.24	226.56 ± 10.72
AF	2T2Hr	522.67 ± 26.50	989.28 ± 48.23	0.444 ± 0.541	0.866 ± 0.153	676.20 ± 22.08	200.94 ± 6.90	321.72 ± 22.77
VF	2T1He	461.68 ± 20.33	146.01 ± 15.90	-0.783 ± 0.397	0.599 ± 0.200	655.88 ± 30.53	154.92 ± 9.21	306.76 ± 16.59
VF	2T2He	439.17 ± 15.12	80.25 ± 18.92	-0.382 ± 0.280	0.595 ± 0.053	753.26 ± 22.75	145.48 ± 4.96	290.16 ± 12.28
VF	2T1Hr	433.87 ± 19.85	821.34 ± 40.54	-0.086 ± 0.420	0.778 ± 0.240	736.15 ± 25.38	185.34 ± 7.58	248.53 ± 15.75
VF	2T2Hr	526.43 ± 21.19	982.40 ± 56.54	0.344 ± 0.556	1.019 ± 0.237	668.56 ± 21.99	207.32 ± 6.33	319.11 ± 17.03

Note. NF = no-feedback condition; AF = auditory feedback condition; VF = vibrotactile feedback condition. 1T = single-target task; 2T1He = unimanual extension task; 2T2He = bimanual extension task; 2T1Hr = unimanual reversal task; 2T2Hr = bimanual reversal task. Values represent estimated marginal means ± standard error (SE).
